# Morphometric Analysis of Dry Human Patella and Patellar Facets

**DOI:** 10.7759/cureus.22879

**Published:** 2022-03-06

**Authors:** Sameen Taj, Gunapriya Raghunath, Karthikeyan Gurusamy, Zareena Begum, Vandana Kaveripakkam, Priya Dharshini

**Affiliations:** 1 Anatomy, Saveetha Medical College, Chennai, IND

**Keywords:** tkr, patella, morphometry, knee joint, articular facets

## Abstract

Background

The patella is also known as the kneecap. It lies in front of the knee joint and protects the joint from damage. It is the largest sesamoid bone in the body and is embedded within the quadriceps tendon. The morphometry of the patella is crucial in forensic analysis, designing of implants, and subsequent reconstruction procedures in the knee as it is a sesamoid bone, without periosteum, whereby the natural healing process becomes difficult. The dimensions of the implant are very crucial for a successful knee replacement procedure. This study aims to provide a comprehensive morphometric analysis of the patella and further compare the same between right and left-sided patella specimens.

Methodology

In total, 50 dry patella specimens, with 26 left-sided specimens and 24 right-sided specimens, were obtained for the study from the Department of Anatomy, Saveetha Medical College, Chennai. The parameters analyzed in the study included height, width, the thickness of the patella, length and width of the articular facets on medial and lateral aspects, and central ridge length.

Results

The morphometric analysis showed the mean height, width, and thickness of patella specimens were 4.07 cm, 4.12 cm, and 2.03 cm, respectively. The dimensions of the articular facet on the lateral aspect were found to show statistical variation compared to the dimensions of the medial articular facet, where p-values of <0.05 were taken as statistically significant. Based on Koyunco’s Classification, 92% of patella specimens were of Type B.

Conclusions

The morphometric analysis of the patella in this study can be helpful in designing implants for reconstruction and for treating orthopedics in patellar reconstruction and fixation procedures.

## Introduction

The patella is the largest sesamoid bone that increases the lever arm and thereby facilitates a 50% increase in quadriceps strength in the extensor apparatus of the knee [1]. Studies on the morphologic and morphometric parameters of the patella are needed as the patella plays a key role in knee biomechanics and subsequent pathophysiology of knee ailments [2].

The patella has two surfaces, namely, anterior and posterior, three borders, and an apex, which is placed distally. Vertical ridges are seen on the anterior surface. The posterior surface has a smooth area and an oval articular area, which is further crossed by a vertical ridge that appears smooth. Patella also shows the presence of two articular facets, namely, medial and lateral, which are usually larger. The facets are divided into three equal parts by horizontal lines which appear faint. An odd small facet in the patellar medial border is separated by a narrow strip. This facet comes into contact only in extreme flexion of the knee joint with the medial femoral condyle [3]. The patella was classified into three categories based on the medial and lateral articular facet width and their curvatures by Wiberg et al. [4]. Based on the width of the medial and lateral width of patellar articular facets, three categories of patella include class A, where both the widths are equal, and class B, where the width of the lateral facet is more than the medial facet. Class B is the most prevalent, as shown by Koyuncu et al. [5]. In the third category, class C, the medial width is more than the lateral width.

The patella bone is involved in different postures such as squatting, sitting, and kneeling, and hence, it is subjected to various modifications depending on the ethnic and cultural practices. Many studies have confirmed ethnic and racial variations of the patella morphometry [6-8].

The patella is used in forensics for identification as it can restraint changes that can occur post-mortem [9]. The morphometric analysis of the patella holds anthropological and clinical importance as it can guide in choosing a patellar implant [5]. When the implant is chosen in case of reconstruction of the patella post-fracture, it leads to the quadriceps lever becoming ineffective, movement restriction, and further leading to early wear and tear in the implant, which progresses to pain and instability in the knee [10].

Most of the existing total knee arthroplasty (TKA) implants are designed to suit the knee anatomy of the Western population. Studies have shown that there are striking variations in knee morphology between Western and Asian populations. In this study, we conducted a morphometric analysis of the patella and classified them based on the width of articular facets in a South Indian population.

## Materials and methods

**Figure 1:**
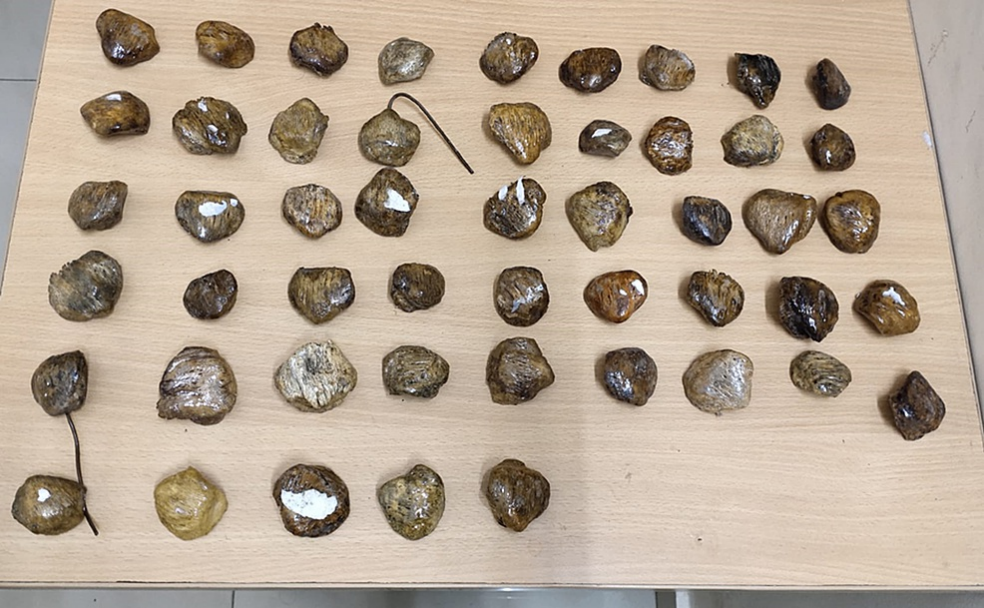
The dry specimens of the patella obtained for the study.

This descriptive cross-sectional study was conducted after receiving approval (SMC/IEC/2021/01/089 dated April 11, 2021) from the Institutional Ethical Review Board (IERB) of Saveetha Medical College, Chennai. The study was commenced in April 2021 and continued for three months. The study include a total of 50 patellae, out of which 26 were right and 24 were left-sided dry patellae of unknown age and sex, which were the available sample size procured from the Department of Anatomy, Saveetha Medical College, Chennai (Figure 1).

Morphologic and morphometric analysis

The morphological classification of the patella is based on Koyuncu’s classification. The patellae were classified as types A, B, and C based on the dimensions of the lateral and medial patellar facets.

**Figure 2:**
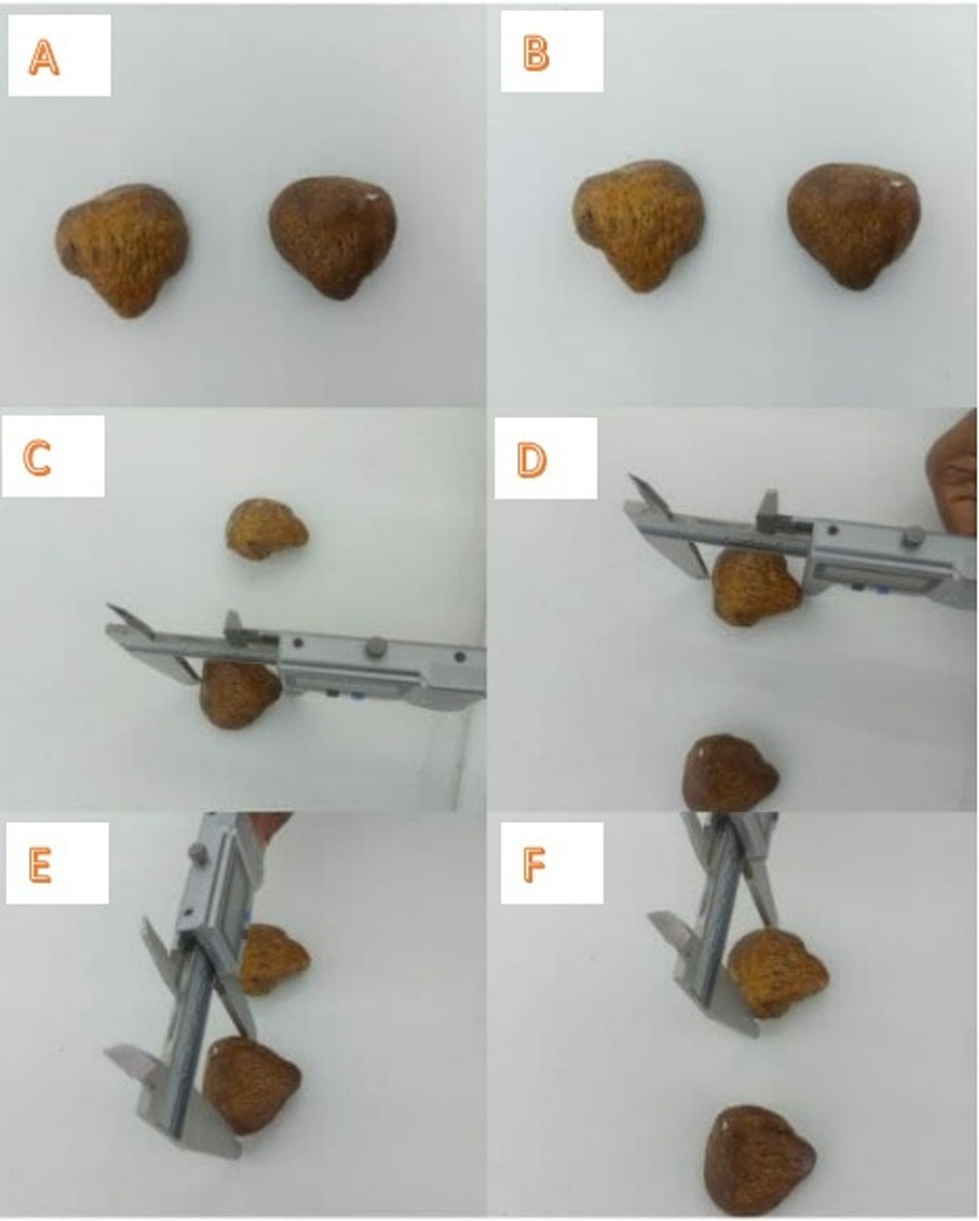
Morphometric analysis of the patella. (A, B) Left and right patella specimens. (C, D) The measurement of the length of patella specimens (linear distance between the superior border and the apex). (E, F) The measurement of the width of patella specimens (linear distance between the medial and lateral border).

**Figure 3:**
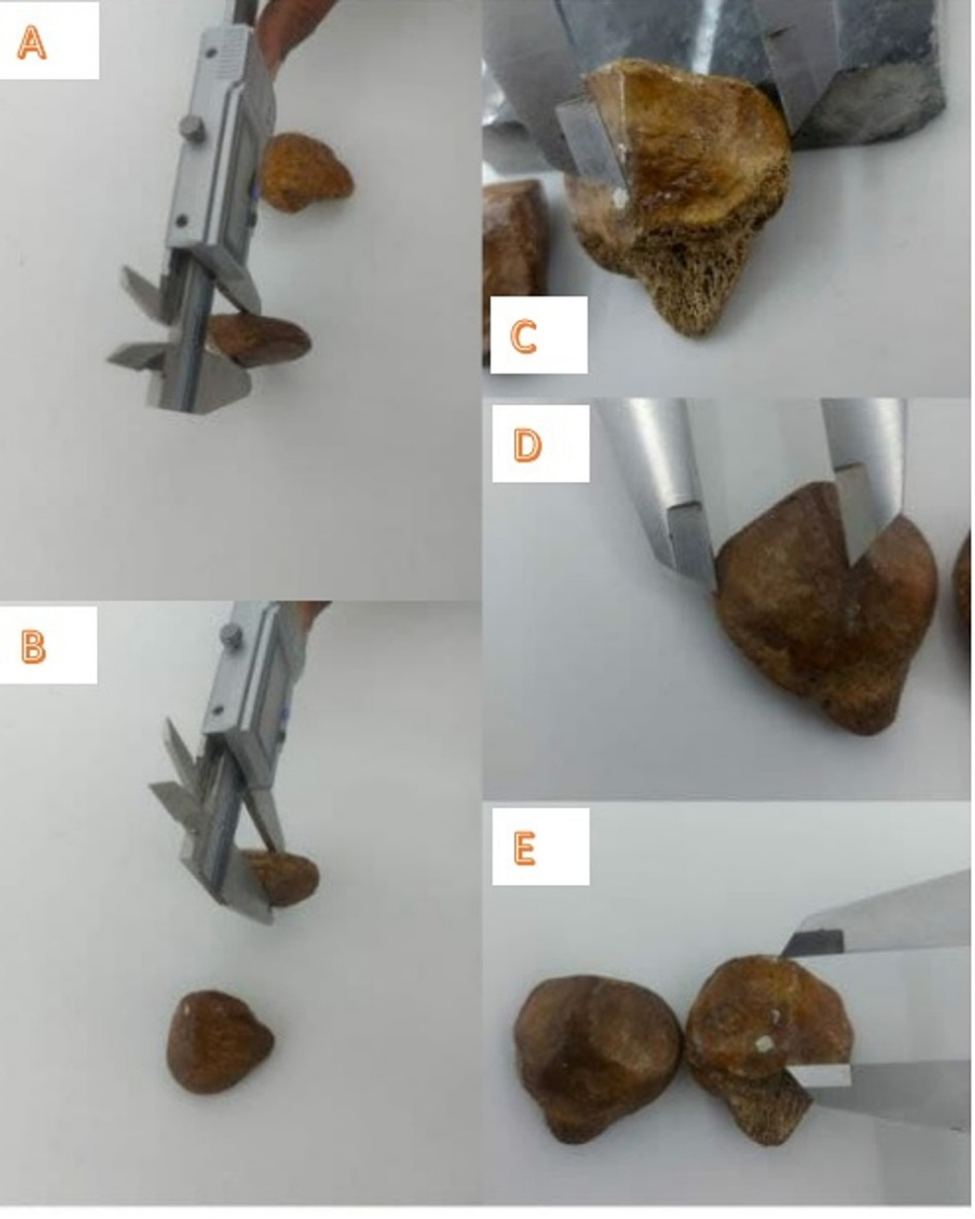
Morphometric analysis of the patella. (A, B) The measurement of the thickness of the patella (linear distance between the anterior surface and the median ridge on the posterior surface). (C) The measurement of the width of the lateral articular facet (maximum width from the lateral border to the median ridge). (D) The measurement of the width of the medial articular facet (maximum width from the medial border to the median ridge). (E) The measurement of the length of the patellar ridge (maximum length from the superior point to the inferior point of the ridge).

The parameters taken for the study included the height, width, and thickness of the patella. The medial and lateral articular facets were analyzed, and the length and width of both facets were measured. The length of the central ridge was also measured [5]. All measurements were taken using digital vernier calipers by the first author of the study. The bony points for these measurements are shown in Figures 2, 3. Specimens with signs of fracture and those showing gross erosions were excluded from the study.

Data collection and analysis

The morphometric parameters measured were tabulated in a Microsoft Excel sheet. As two groups of right and patella measurements were compared, Student’s t-test was used to determine the statistical difference in the morphometry of patellar specimens using SPSS software version 23.0 (IBM Corp., Armonk, NY, USA). P-values of <0.05 were considered statistically significant.

## Results

The patella specimens were measured, and the morphometric parameters were assessed. The results of the descriptive statistical analysis including the mean, maximum values, minimum values, and standard deviation (SD) of right and left-sided patella specimens are shown in Table 1. P-values of <0.05 were considered statistically significant.

**Table 1:** The mean ± SD, minimum values, and maximum values along with t-values and p-values of different parameters of right and left-sided specimens of the patella (in cm). SD: standard deviation

Parameters	Right patella (N = 24)			Left patella (N = 26)			t-value	P-value
	Mean ± SD	Minimum	Maximum	Mean ± SD	Minimum	Maximum		
Patella height	4.00 ± 0.34	3.50	4.80	4.13 ± 0.49	3.30	5.10	-1.111	0.272
Patella width	4.11 ± 0.37	3.30	4.90	4.13 ± 0.28	3.60	4.50	-0.285	0.777
Patella thickness	2.04 ± 0.16	1.80	2.40	2.01 ± 0.18	1.70	2.40	0.622	0.537
Ridge length	2.77 ± 0.23	2.30	3.10	2.75 ± 0.35	1.90	3.30	0.201	0.842
Medial articular facet length	2.42 ± 0.24	2.00	3.00	2.41 ± 0.29	1.90	3.00	0.177	0.860
Medial articular facet width	2.15 ± 0.21	1.80	2.50	2.05 ± 0.26	1.50	2.60	1.528	0.133
Lateral articular facet length	2.92 ± 0.19	2.30	3.20	2.90 ± 0.28	2.30	3.50	0.188	0.852
Lateral articular facet width	2.43 ± 0.19	2.10	2.70	2.48 ± 0.20	2.10	2.90	-0.989	0.328

No statistically significant difference was found in parameters between the right and the left patella, as can be seen in Table 1, except the mean patellar height was slightly greater on the left side.

The medial and lateral articular facet widths were compared on both right and left sides and showed statistical significance on both sides (Tables 2, 3).

**Table 2:** The mean values of the medial and lateral articular facet width of right-sided patella specimens along with statistical significance.

Variables	N	Mean ± SD	t-value	P-value
Medial articular facet width right	24	2.16 ± 0.19	-6.922	0.0001
Lateral articular facet width right	24	2.41 ± 0.18		

**Table 3:** The mean values of the medial and lateral articular facet width of left-sided patella specimens along with statistical significance.

Variables	N	Mean ± SD	t-value	P-value
Medial articular facet width left	26	2.05 ± 0.28	-7.590	0.0001
Lateral articular facet width left	26	2.50 ± 0.20		

The dimensions of the articular facets on the medial and lateral aspects of the patella showed statistical differences (Tables 2, 3). It was found that the lateral facet had a larger width compared to the medial facet.

Table 4 shows the classification of the patella according to Koyuncu’s classification, where patellae are classified according to the dimensions of the articular facets in the medial and lateral aspects. The majority of patellae in this study belonged to class B, with 46 specimens, whereas two were class A and two were class C.

**Table 4:** The classification of the patella in this study based on Koyuncu’s classification. Class A: where widths of both articular facets are the same; class B: where the articular facet on the lateral aspect is larger than the medial facet; class C: where the articular facet on the medial aspect is larger than the lateral facet.

Class	Number of patellae (%)
A	4%
B	92%
C	4%

## Discussion

The patella plays multiple roles in optimizing the movements of the knee joint. It acts as a primary mechanical pulley, increases the lever arm, and influences the change in the direction of the pull of the quadriceps muscle, which is effective in the range of motion of the knee joint. Huberti and Hayes stated that the patella plays a crucial role in the final range of knee extension at the range of 30° to 0° [11]. Moreover, 31% of the total extension torque of the knee was contributed by the patella when the knee was fully extended, against the contribution of only 13% when the knee was flexed in the range of 90° to 120°. In addition, the patella also functions as a shield in the anterior aspect, thereby increasing friction that can occur between the condyles of the femur and the tendon of the quadriceps [12].

Most of the existing TKA implants are designed to suit the knee anatomy of the Western population. Studies have shown that there are striking variations in knee morphology between Western and Asian populations [13]. The implants for arthroplasty and surgical fixation in fractured patella can be customized according to the morphometry.

The values of the mean height, thickness, and width of the patella in this study were 4.07 cm, 2.03 cm, and 4.12 cm, respectively, which was less compared to the studies done in South African, Korean, Western, and Chinese populations, as shown in Table 5. The differences in the measurement between Asian and Western populations show that surgeons in Asia can customize specific implants based on the morphometry, and surgical techniques can be confirmed by the variations in morphometry which can eventually lead to better functional and clinical outcomes and patient satisfaction [14,15]. The thickness is the least in the Indian population, and the width is lower in the Chinese and Malaysian populations compared to our study. Patellar thickness remains one of the most pertinent factors for a successful surgical outcome. The morphometric values of the current study and their comparison with previous studies are shown in Table 5.

**Table 5:** A comparison of the morphometric values of this study with studies done in other geographic populations.

Study	Mean patellar height (cm)	Mean patellar thickness (cm)	Mean patellar width (cm)
Present study (2022); South Indian population	4.07	2.03	4.12
Olateju et al. (2013) [16]; South African population	4.37	2.41	4.51
Yoo et al. (2007) [17]; Korean population	4.46	2.23	4.41
Iranpour et al. (2008) [9]; United Kingdom	3.43	2.24	Not available
Baldwin and House (2005) [18]; Western population	Not available	2.26	4.69
Shang et al. (2014) [19]; Chinese population	3.99	2.27	4.41
Katchy et al. (2020) [20]; South-East Nigeria	4.61	2.66	4.69
Rahman et al. (2020) [21]; Malaysian population	3.13	2.07	4.07

Table 6 shows the comparison of morphometric values of this study with other studies conducted in India.

**Table 6:** A comparison of morphometry of the patella with other Indian studies.

Study	Patella height (cm)	Patella width (cm)	Patella thickness (cm)	Width of the medial articular facet (cm)	Width of the lateral articular facet (cm)
Present study (2022)	4.07	4.12	2.03	2.10	2.46
Muhamed et al. (2017) [22]; South Indian population	Not available	4.22	2.03	Not available	2.25
Biswas et al. (2019) [23]; Eastern Indian population	3.99	4.08	1.95	1.51	2.25
Kumar et al. (2020) [24]; North Indian population	Not available	4.42	1.93	Not available	2.50
Baisakh et al. (2021) [25]; East Indian population	3.67	3.65	1.83	1.87	2.10

In this study, there were no significant differences in the right and left sides of the patella which is similar to other previous studies. These parameters will undoubtedly be of immense benefit to the manufacturers of prostheses who had based their sizes on Western parameters, which may not be suitable for the Indian population [5]. The data presented in this study can help in the manufacture of implants in arthroplasty procedures of the knee joint or in gross comminuted fracture of the patella for the knee joint to function optimally [21].

The dimensions were low in an East Indian population, and higher values were found in a North Indian population compared to a South Indian population, as shown in this study [22-25]. Although the reasons for these variations cannot be validated, the differences may be due to the differences in the method of measuring, age, sex, stature, and body mass index. 

The scope of the study could have been broadened by increasing the sample size and including specimens of diverse ethnicities. Some of the limitations of this study include its small sample size and the fact that the selected specimens were dry specimens where the age and sex of the specimens were unknown. No known variations were observed in this study consistent with other studies.

## Conclusions

This study showed that the mean height, width, and thickness were 4.07 cm, 4.12 cm, and 2.03 cm, respectively. These findings are consistent with other Indian studies, as shown in Table 6. The data presented in this study can be helpful in the forensic analysis and ethnic morphometry of patella specimens. This investigation has determined the important dimensions of the patella bone in a South Indian population which can provide guidelines to manufacture prosthetic implants of the patella which is clinically essential because the South Indian population had smaller anthropometric measurements of the patella compared with the North Indian Population, but larger dimensions when compared with an East Indian Population. Hence, this study can help in successful treatment planning in cases of anterior knee pain and patellofemoral joint disorders requiring surgical procedures such as TKA.
